# Interactions of Cisplatin and Daunorubicin at the Chromatin Level

**DOI:** 10.1038/s41598-020-57702-7

**Published:** 2020-01-24

**Authors:** Erfaneh Firouzi Niaki, Thibaut Van Acker, László Imre, Péter Nánási, Szabolcs Tarapcsák, Zsolt Bacsó, Frank Vanhaecke, Gábor Szabó

**Affiliations:** 1 Department of Biophysics and Cell Biology, University of Debrecen, Faculty of Medicine, Debrecen, H-4032 Hungary; 2 Department of Chemistry, Atomic & Mass Spectrometry – A&MS Research Unit, Ghent University, Campus Sterre, Krijgslaan 281-S12, 9000 Ghent, Belgium

**Keywords:** Nucleosomes, Chromatin structure, Chemotherapy

## Abstract

Unexpectedly, the widely used anticancer agents Cisplatin (Cis-Pt) and Daunorubicin (Dauno) exhibited cell type- and concentration-dependent synergy or antagonism *in vitro*. We attempted to interpret these effects in terms of the changes elicited by the drugs in the chromatin, the target held primarily responsible for the cytotoxicity of both agents. We measured the effect of Cis-Pt on the levels of Dauno in different cell compartments, the effect of Cis-Pt on Dauno-induced nucleosome eviction, and assessed the influence of Dauno on DNA platination in flow- and laser scanning cytometry as well as in laser ablation-inductively coupled plasma-mass spectrometry assays. We show that the two drugs antagonize each other through a decrease of interstrand crosslinks upon co-treatment with Dauno, and also via the diminished Dauno uptake in the presence of Cis-Pt, and both effects are observed already at low Dauno concentrations. At high Dauno concentrations synergy becomes dominant because histone eviction by Dauno intercalation into the DNA is enhanced in the presence of co-treatment with Cis-Pt. These interactions may have an impact on the efficacy of combination treatment protocols, considering the long retention time of DNA adducts formed by both agents.

## Introduction

Anticancer drugs that target DNA are some of the most effective agents in combating cancer. Anthracyclines and platinum-based drugs have been exploited in combination chemotherapy to treat a broad variety of different types of cancer, such as ovarian carcinoma^1,2^, small cell lung cancer^3^, and in endometrial carcinoma^4^, among others. Although many of the cellular targets of these anticancer agents are known, their involvement in toxicity is poorly understood. Therefore, how they interact with each other when used in combination is difficult to predict.

Daunorubicin (Dauno)^5^, the first discovered anthracycline compound^6^, affects a broad range of biochemical processes and a number of different mechanisms have been proposed to be responsible for Dauno-elicited cytotoxicity. These include inhibition of DNA and RNA synthesis (mainly due to binding of the drug to the DNA), Topoisomerase II poisoning (by trapping the enzyme at cleavage sites), oxidative stress (due to formation of reactive oxygen species), involving also lipid peroxidation (by chelating iron), as reviewed in^7^. Dauno binds the H1 family of histones and causes chromatin aggregation thereby influencing higher-order chromatin conformation^8^. As a DNA intercalator, Dauno unwinds the double-stranded DNA by 8° per each intercalated molecule^8,9^, thereby extending it, and also increasing its melting temperature^10^. Dauno will relax, and at higher concentrations overwind the DNA^9,11^. Simultaneously with relaxation, nucleosomes become evicted^9,12^. Thus there is ample reason to assume that the binding of other DNA targeting drugs may be altered when Dauno is simultaneously applied influencing DNA topology. Since Dauno and many other DNA targeting drugs such as Cisplatin (Cis-Pt) form rather stable adducts in the DNA^13^, they may mutually influence each other’s intracellular distribution, binding and effects even in sequential treatment regimens.

The DNA binding of Cis-Pt, one of the most potent antitumor agents, is known to be sensitive to DNA conformation in at least one of its binding modes^14^. Cis-Pt interacts with DNA, RNA as well as with proteins. Since the ratio of Cis-Pt modified molecules to the total number of the same molecular species in the cell is much higher in the case of DNA than for the other classes of molecules, binding of this agent to the DNA is generally perceived as the main cause of its toxicity^15^. The Cis-Pt-DNA complexes form primarily at the internucleosomal linker regions^16^. The relative amount of the different adducts is determined by the kinetics of formation, their chemical stability, as well as by their removal executed by the repair machinery. 80–90% of all DNA adducts formed by Cis-Pt comprises intrastrand crosslinks, primarily forming between the adjacent purines, while the frequency of interstrand crosslinks (ICLs) between the complementary strands is about 1–5%. As an example, 48,000 Pt-DNA adducts per cell gave rise to 50% inhibition of cell growth while the number of ICLs was estimated only 480/cell^17^. The frequency of the DNA and protein monoadducts, forming without any cross-linking, is relatively negligible^18^. The contribution of the different adducts to toxicity has not been fully clarified. ICLs are considered not to be the main cytotoxic lesions based on the observation that transplatin, although much more prone to form ICLs than Cis-Pt, is clinically ineffective [19]. However, the increased sensitivity of Fanconi anaemia cells (defective in the repair of ICLs), to ICL-forming agents suggests that their contribution to cytotoxicity may be significant^19^. Importantly for the current study, ICL formation of Cis-Pt^20^, and also of another interstrand cross-linker, psoralen^21^, are favored in negatively supercoiled as compared to linear or relaxed DNA. On the other hand, bending of DNA is required for the formation of intrastrand crosslinks^22,23^. These observations together raise the possibility that alterations in DNA topology, necessarily accompanying the co-administration of DNA intercalators, may result in major consequences on Cis-Pt toxicity. Conversely, Cis-Pt-elicited crosslinks may fix internucleosomal DNA in constrained conformation antagonizing intercalation.

In view of these complexities, we set out to determine how the Dauno evoked changes in chromatin structure and DNA topology affect the formation of Cis-Pt-DNA adducts, and how Cis-Pt influences Dauno uptake and binding. We applied laser scanning cytometric (LSC) and flow-cytometric assays of the intracellular, intranuclear and DNA-bound Dauno, and of the Cis-Pt-DNA adducts. Histone eviction was measured by Quantitative Imaging of the Nuclei after Elution with salt/intercalator (QINESIn) developed in our lab^12^. The nuclear comet assay based on the preferential reassociation of alkali-denatured genomic DNA derived from Cis-Pt-treated cells^24,25^, modified to improve its specificity and throughput, was used to monitor ICL formation. Quantitative laser ablation-inductively coupled plasma-mass spectrometry (LA-ICP-MS) spot analysis was applied for the determination of Pt (at Ghent University, Belgium), yielding absolute numbers for total Cis-Pt-DNA adducts and ICLs per nucleus. These studies were performed in an experimental system where Dauno and Cis-Pt either antagonize or synergize each other in a cell-type and concentration dependent manner.

## Results

### Cis-Pt and Dauno synergize or antagonize each other in a cell-type and dose dependent manner

**Figure 1:**
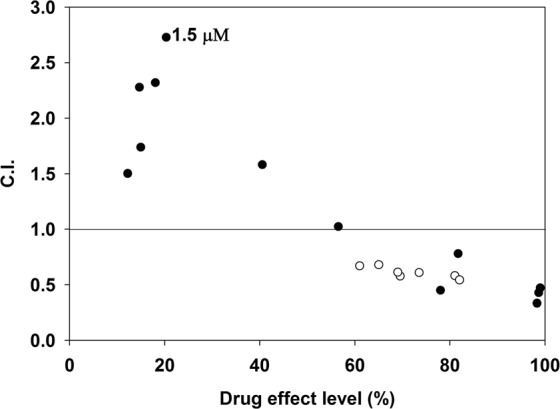
Combination Index plot of Cis-Pt and Dauno treatment of HeLa and Jurkat cells. HeLa (●) and Jurkat (°) cells were co-treated with 40 μM Cis-Pt and different concentrations of Dauno for 16 h, and the viability of the cells was measured by the Resazurin assay. The dose-effect profiles were used to calculate Combination Index (C.I.) values as described in the Materials and Methods section. The data points below or above the line indicate synergistic or antagonistic drug interactions, respectively, and represent averages of three independent experiments. The dose-response data of the averages are shown in Fig. S1. The results of the pre-titration of Cis-Pt doses performed in Jurkat cells, what had led us to use 40 μM of this drug throughout the experiments, are shown in Fig. S2. The toxicity values obtained at the different Dauno concentrations combined with a constant dose of Cis-Pt are represented on the X axis (drug effect levels). The matching values of C.I. and Dauno concentrations are shown in supplemantay Table 2. The concentration of Dauno where the highest degree of antagonism was observed (1.5 µM) is indicated on the Figure.

Synergy between two drugs (when the observed effect is greater than the product of the effects of each individual agent), vs. antagonism (when the observed effect is smaller than what is expected when the effects add up), are not readily determined by comparison of the individual effects with those of the combinations (Fig. S1), due mainly to the non-linearity of the dose-effect relationships. Therefore, we used the combination index (C.I.) method based on the median-effect principle of the mass-action law^26^, performed according to Chou-Talalay (see also Materials and Methods), to determine the mode of drug interaction. Cytotoxicity was evaluated by performing a Resazurin cell viability assay. Our initial measurements of cytotoxic interactions between Dauno and Cis-Pt added together to Jurkat cells confirmed the general synergism of the two drugs over a wide concentration ratio, what is expected in view of clinical experience^1,2^ exhibited best at 40 uM Cis-Pt for both low and high Dauno concentration (Fig. S2). Unexpectedly, in sharp contrast with the Jurkat cells, in HeLa cells antagonism was observed (C.I. > 1) at the subtoxic Dauno concentration range of 0.3–1.8 μM, and synergism (C.I. < 1) at toxic Dauno concentrations ranging between 3–12 μM, using 40 μM Cis-Pt (Fig. 1 and supplementary Table 2). The phenomenon of dose- and cell line-dependent synergism vs. antagonism is not unique to Jurkat and HeLa cells (e.g. T47D cells exhibited antagonism of the two drugs at high, and synergy at low Dauno concentration; data not shown). It was also not unique to Dauno, since Doxorubicin (Dox), another anthracycline closely related to Dauno also showed such a varied response (data not shown). We set out to investigate the possible scenarios of interactions that may lead to synergism or antagonism.

### Co-treatment with Cis-Pt differentially alters the amount of total cellular, chromatin-bound and DNA-bound Dauno

**Figure 2:**
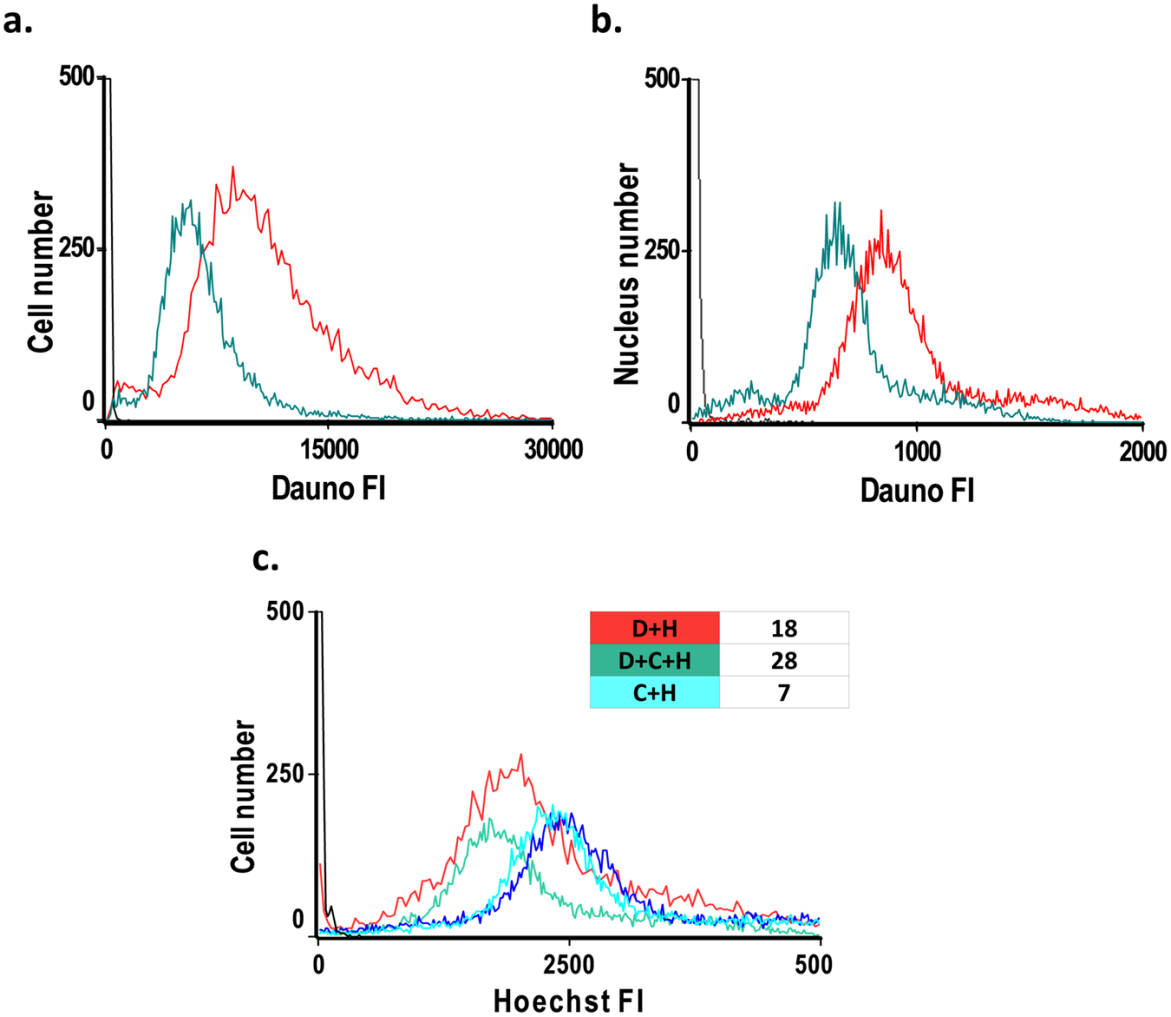
Effect of Cis-Pt on Dauno uptake. Total cellular (**a**) and nuclear (**b**) fluorescence intensity distributions of Dauno, and of Hoechst 33342 (**c**), measured by flow-cytometry. Fluorescence intensity distributions of 20,000 events were recorded in a representative experiment. (**a,b**) Distribution curves of Dauno fluorescence of HeLa cells (**a)**, or nuclei (**b)**, after treatment of live cells with 1.5 μM Dauno only (red line), with 1.5 μM Dauno in combination with 40 μM Cis-Pt (green), or left untreated (black). (**c**) Hoechst 33342 fluoresence intensity distributions of live HeLa cells. The Hoechst dye (H) was added to the cells at 35 μM concentration following incubation with Dauno and/or Cis-Pt at different combinations: Dauno and Hoechst (red), Dauno, Cis-Pt and Hoechst (green), Hoechst 33342 only (blue), Hoechst 33342 and Cis-Pt (turquoise). The table inset shows the decrease of the mean Hoechst fluorescence in the samples co-treated with Dauno (D + H), Dauno + Cis-Pt (D + C + H) and Cis-Pt only (C + H), in % of the control (blue).

We first evaluated whether the antagonism between the two drugs might be related to an effect of Cis-Pt on the amount of Dauno taken up into the cells, accumulating in the acidic endosomal compartment^27^, by assessing Dauno fluorescence in live cells using flow-cytometry. In parallel, the total nuclear fluorescence of Dauno, accounting for the chromatin-bound drug, was also measured. Dauno fluorescence was significantly decreased in Cis-Pt-Dauno co-treated cells as compared to cells incubated with Dauno only, as measured both in live cells (Fig. 2a) and nuclei (Fig. 2b). Since Dauno also binds to histones^28^ in addition to DNA, we performed an assay to directly measure the effect of Cis-Pt co-treatment on DNA-bound Dauno in live cells via the quenching of Hoechst 33342 fluorescence^29^. Hoechst 33342 enters live cells and becomes brightly fluorescent upon binding through the minor groove of the DNA to tandem AT base pairs^30–32^. Its green emission is absorbed by Dauno quenching Hoechst fluorescence what can be utilized as a measure of DNA-bound anthracycline. The decrease of Hoechst fluorescence in the presence of Dauno was stronger when Cis-Pt was also added. This difference can be partly accounted for by the influence of Cis-Pt on Hoechst fluorescence (see Table inset of Fig. 2c). However, an increased level of DNA-bound anthracycline in the presence of Cis-Pt co-treatment was corroborated in experiments when the DNA of cells treated with Dox (an anthracyclin closely related to Dauno) was isolated, run on agarose gels and visualized by ethidium bromide (EBr) staining, see Fig. S3). Thus, in view of the fact that DNA-bound anthracycline levels certainly did not decrease (rather increased based on Fig. S3), the decreased nuclear fluorescence of Dauno in the presence of Cis-Pt (Fig. 2b) may reflect a significantly decreased presence of histone-bound Dauno levels, likely due to histone eviction elicited by the anthracycline. In summary, Cis-Pt decreases the amount of anthracycline accumulating in the cytoplasmic acidic compartment (comprising the dominant species of the drug), it also decreases the amount of the histone-bound Dauno, while the amount of DNA-bound anthracycline increases.

### Cis-Pt facilitates Dauno-induced histone eviction

**Figure 3:**
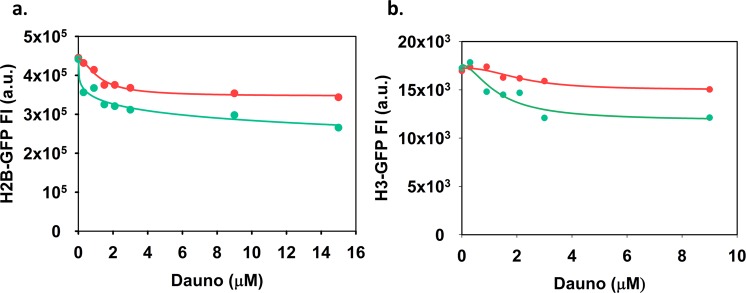
Effect of Cis-Pt on Dauno-induced histone eviction. (**a**) QINESIn assay of histone eviction^12^. Nuclei of H2B-GFP-expressor HeLa cells were treated with a concentration series of Dauno only (red) or with Dauno in combination with Cis-Pt (green). The GFP fluorescence intensity of the individual nuclei was recorded by LSC. (**b**) Live H3-GFP-expressor HeLa cells were treated with Dauno only or with Dauno in combination with Cis-Pt (red and green line, respectively). Subsequently nuclei were prepared and their histone-GFP levels measured by LSC. The mean values of ~1,000 nuclei were plotted as a function of the Dauno concentration used. For both panel **a** and **b**, the means of the fluorescence distribution curves, recorded for the samples treated and run in parallel, are plotted. Representative distribution curves of the experiment are shown in Fig. S4. Fluorescence intensities are shown in arbitrary units (a.u.).

A newly discovered effect of anthracyclines, also implicated in their cytotoxicity, is induction of nucleosome eviction due to intercalation^9,12^. We investigated whether this effect may be influenced by Cis-Pt. When live cells or isolated nuclei were co-treated with the intercalator and Cis-Pt, the intercalator-induced eviction of the histone dimers (Fig. 3a), as well as of tetrasomes (Fig. 3b) was substantially augmented. Thus, the amount of histone-bound Dauno is expected to decrease in the nucleus when cells are co-treated with Dauno and Cis-Pt, apparently in the entire concentration range of the intercalator.

### Detection of ICLs using a modified alkaline comet assay

**Figure 4:**
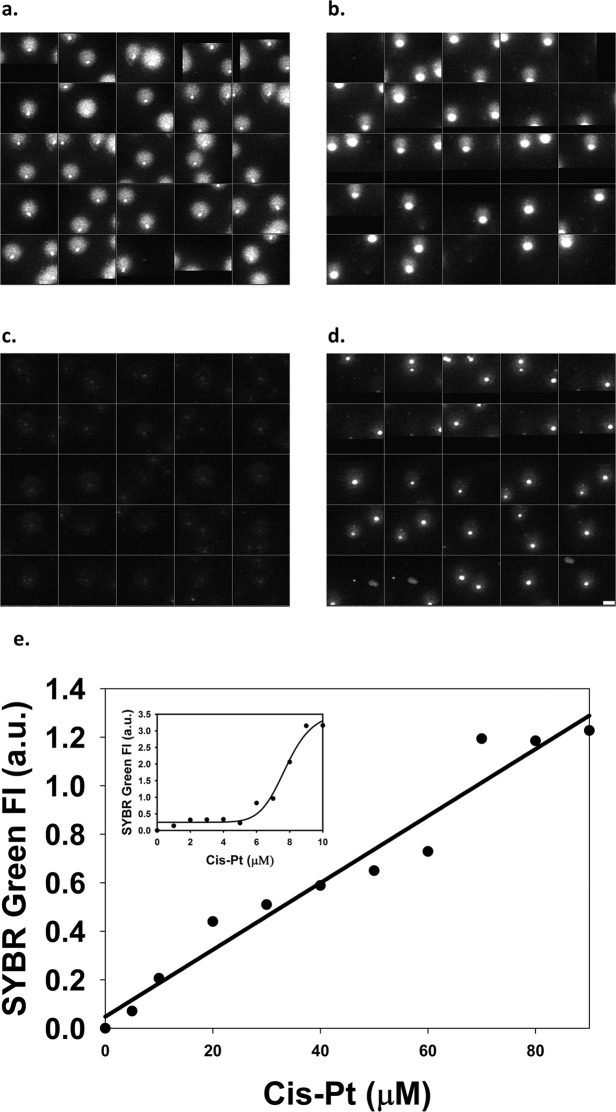
Measurement of ICLs using the alkaline comet assay combined with S1 endonuclease treatment and SYBR Green I staining. (**a,c)** Alkaline comets of Jurkat cells in the absence of drug treatment. (**b,d)** Alkaline comets after treatment with 40 μM Cis-Pt. The effect of S1 endonuclease treatment performed after renaturation is shown in (**c**) and (**d**). Scale bars = 10 μM. (**e**) Jurkat cells were treated with different concentrations (1–90 μM) of Cis-Pt, and the mean fluorescence intensities of the renatured comets were recorded by LSC after being digested with S1 endonuclease and stained with SYBR Green I. The median values of the fluorescence intensity distributions of ~400 nuclei were plotted as a function of Cis-Pt concentration. ICL levels measured in the lower concentration range of Cis-Pt in a separate experiment are shown in the inset. Fluorescence intensities are shown in arbitrary units (a.u.).

Another possible mode of interaction between the two classes of drugs is relaxation of supercoiling by the intercalator, mitigating Cis-Pt-DNA ICL formation^20^. In order to study this possibility, we employed a modified version of the alkaline comet assay where the ICL-dependent increment in the renaturation of alkali-denatured samples of nuclei is measured^24^. In view of the rather high background of renatured DNA in the absence of crosslinker in that procedure (Fig. 4a), we used S1 nuclease treatment to remove any non-renatured DNA, and the SYBR Green I dye to stain ds DNA (Fig. 4c,d). Using these modifications and employing LSC to measure the fluorescence of nuclear comets (or, with equivalent results, nuclear halos; see Materials and Methods), ICL detection was made possible in a wide concentration range of Cis-Pt added to live cells, above ~5 μM (Fig. 4e). In the subsequent experiments we used this modified assay to detect ICLs.

### Co-treatment with Dauno influences Cis-Pt-DNA adduct levels

**Figure 5:**
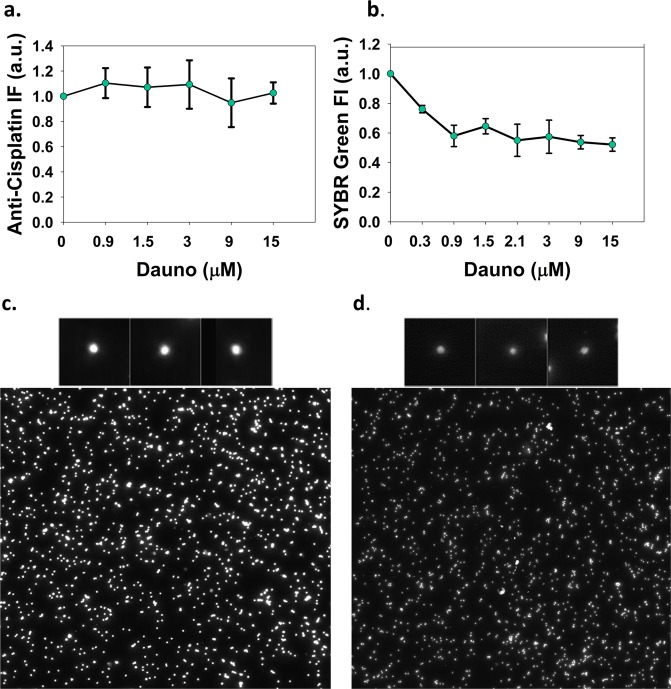
Effect of Dauno treatment of nuclei on the formation of Cis-Pt-DNA adducts. Agarose-embedded nuclei of HeLa cells co-treated with 40 μM Cis-Pt and Dauno used at the concentrations indicated, for 16 hrs (**a**,**b**). The amount of all Cis-Pt-DNA adducts (**a**) and ICLs (**b**) were measured by LSC. (**a**) Total Cis-Pt-DNA adduct levels detected by indirect immunofluorescence using anti-Cisplatin primary and Alexa 488 conjugated secondary antibody on deproteinized nuclear halo samples. (**b**) ICL levels measured by SYBR Green I staining of double-stranded DNA. The nuclear halos were deproteinized after renaturation of the alkaline-denatured DNA strands and the undenatured DNA molecules were removed by S1 nuclease treatment. (**c**,**d**) Field-images of the LSC scans of alkaline halo samples in the case of nuclei treated with Cis-Pt only (**c**), or with Cis-Pt and 15 μM Dauno (**d**). Fluorescence intensities are shown in arbitrary units (a.u.). Representative nuclei of each sample are shown in the insets. Error bars in panel **a** and **b** represent SEM values calculated based on two independent biological experiments, each in duplicate.

In order to assess the effects of Dauno intercalation on the formation of Cis-Pt-DNA adducts, nuclei of HeLa cells were exposed to combinations of 40 μM of Cis-Pt and Dauno used at various concentrations. ICL levels were assessed employing the modified comet assay described above (Fig. 4), while total Cis-Pt DNA adducts were measured via immunofluorescence using an antibody recognizing all Cis-Pt-DNA adducts. We observed no change in the level of total DNA-Pt adduct levels (Fig. 5a–c), while there was a significant reduction in ICL formation (Fig. 5b–d). Notably, this effect was detected already in the low concentration range of the intercalator.

### Effect of nucleosome eviction on Cis-Pt-DNA adduct levels

**Figure 6:**
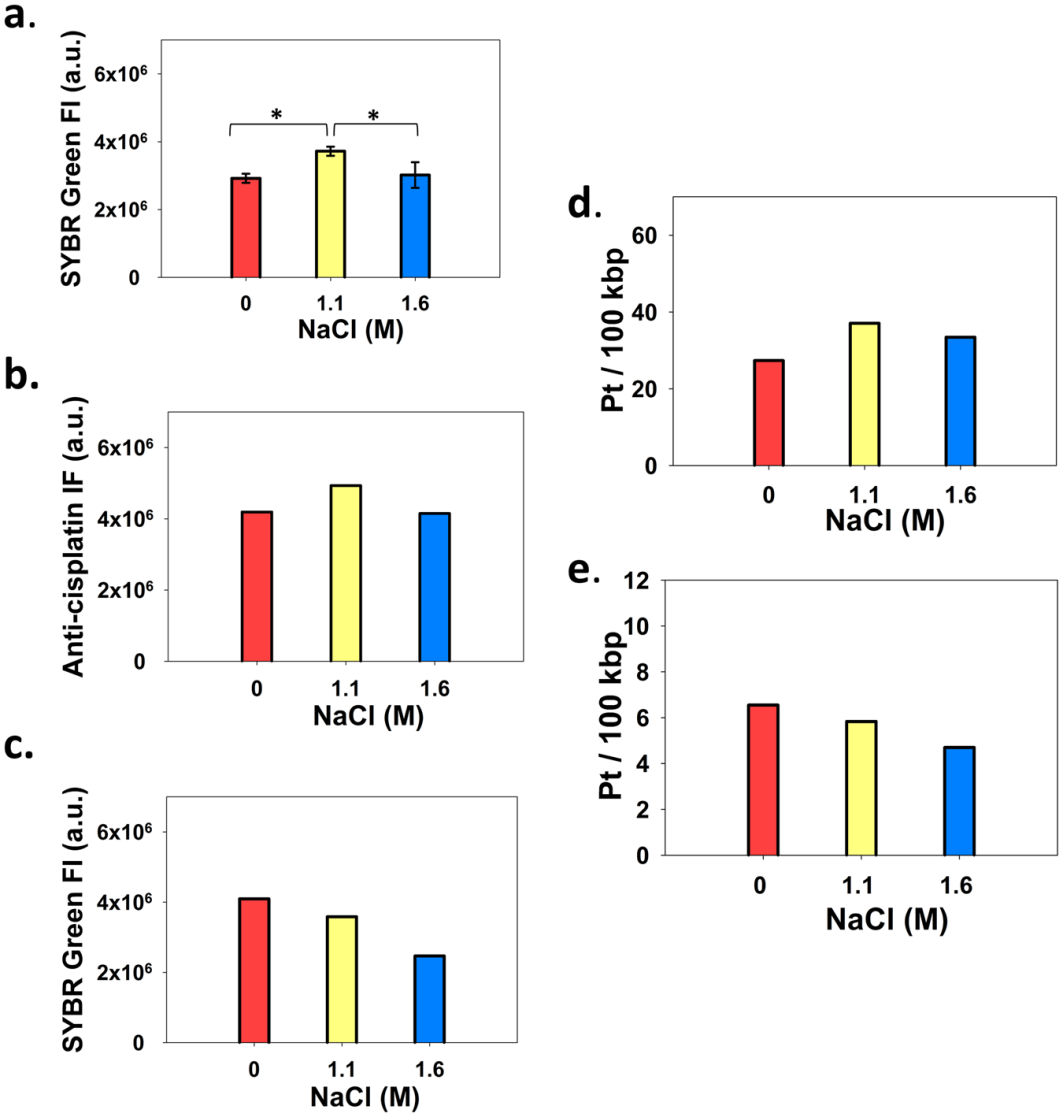
Effect of histone eviction on the formation of DNA-Cis-Pt adducts. Total Cis-Pt-DNA adduct (**a**,**b, d**) and ICL levels (**c**,**e**) formed in HeLa nuclei after pre-treatment with 1.1 M and 1.6 M of NaCl, or with PBS only (indicated as 0), followed by incubation with 40 μM Cis-Pt for 16 h. (**a–e**) Total Cis-Pt-DNA adduct levels were measured (**a**) by SYBR Green staining of the renatured nuclear halos (without S1 nuclease treatment), by immunofluorescence (**b**, showing the median values of the fluorescence distributions) as in Fig. 5, or by LA-ICP-MS (**d**). ICLs were measured after S1 endonuclease digestion of the renatured, deproteinized halo samples, by SYBR Green staining (**c**) or by ICP-LA-MS (**e**). Fluorescence intensities are shown in arbitrary units (a.u.). In (**d**) and (**e**) the absolute number of Pt atoms per 100 kbp of DNA are shown. Error bars denote SD of the means of 4 independent experiments (**a**). One of the samples represented by panel **a** and the same sample shown in **c** were also measured by ICP-LA-MS (panels **d** and **e**, respectively). Statistical calculation for panel **a** was by two-sided t-test. * designates statistical significance at p < 0.02. Representative distribution curves for panels **b** and **c** are shown in Fig. S5. Fluorescence intensities are shown in arbitrary units (a.u.).

Dauno can cause an increase of total Cis-Pt-DNA adduct levels as well as of ICLs due to increased accessibility of the DNA in the wake of nucleosomal eviction. ICL levels may be increased just because total adduct levels are increased. On the other hand, they can be decreased as a result of the relaxation of the superhelical twist accompanying intercalation^12^ and also as a result of nucleosome eviction allowing for free twist-writhe interconversion. In view of the complexity of possible scenarios of drug interactions, a model system was set up to learn how nucleosome eviction, in the absence of any other effects of Dauno, might influence Cis-Pt binding to the DNA. We measured total Cis-Pt adduct levels and ICLs in nuclei pre-treated with salt to remove the dimers (1.1 M NaCl), or all the histones (1.6 M NaCl). Total adduct levels were assessed by three independent methods: by immunofluorescence using the anti-Cis-Pt antibody (Fig. 6b), by measuring the fluorescence of ds DNA in the renatured samples when S1 digestion step was omitted so as to detect all the Cis-Pt adducts renaturing into ds DNA when (any) two strands are covalently linked by Cis-Pt (Fig. 6a), and also by LA-ICP-MS (Fig. 6d). ICLs were measured by the modified comet assay described above (Fig. 6c) as well as by LA-ICP-MS following the treatments performed in the comet assay (Fig. 6e). As Fig. 6 shows, the different approaches gave similar results. Total DNA platination was increased at intermediate salt levels only, while ICLs were decreased, moderately at 1.1 M, and more strongly at 1.6 M salt concentration.

## Discussion

We have observed a cell type- and dose-dependent interaction between Dauno and Cis-Pt (see Fig. 1 and supplementary Table 2), which warrants a detailed analyses of the mechanistic components of the interactions in the case of their combined application in cancer chemotherapy. Reminiscent of our findings, cell type and dose-dependent synergism vs. antagonism was described for the combination of Cis-Pt with a deoxycytidine analogue^33^. In view of the fact that both Dauno and Cis-Pt appear to primarily target the chromatin^5,15^, we set out to assess how they affect each other’s binding to particular cellular components, focusing on the chromatin. There is little doubt that the mode of Cis-Pt action most relevant to cytotoxicity is its binding to the DNA^15^. Although the effects of Dauno are more diverse, its intereaction with the different components of the chromatin are generally considered of primary importance for its toxicity^7^. Also in line with the hypothesis that Dauno exerts its cytotoxicity mainly in the nucleus, a shift in its cellular distribution was observed toward higher cytoplasmic/nuclear ratios in resistant cells^34^.

Regarding the effect of Cis-Pt co-treatment on Dauno levels (Fig. 2a), total Dauno fluorescence representing mainly the acidic endosomal compartment where amphiphylic compounds spontaneously accumulate, decreases upon co-treatment. Therefore, if extranuclear effects are also important factors in Dauno toxicity, Cis-Pt could antagonize them by reducing the cytoplasmic Dauno levels. The mechanism of decreased uptake was not further investigated in this study focusing on possible chromatin level interactions. Cis-Pt also decreased total nuclear Dauno fluorescence (Fig. 2b). Dauno binds more avidly to DNA than to histones^35^, but its fluorescence is quenched only by DNA^36^. Therefore, the fluorescence intensities measured probably reflect the presence of both Dauno-DNA and Dauno-histone complexes, but in an unknown ratio. Therefore, we applied an assay that can directly measure DNA-bound Dauno, based on the quenching of Hoechst 33342 fluorescence by Dauno when they are in molecular proximity^29^. The data of Fig. 2c and Fig. S3 reveal that the levels of DNA-bound Dauno do not decrease (Fig. 2c), rather increase (Fig. S3) upon Cis-Pt co-treatment, so the diminished total nuclear fluorescence is likely due to decreased amounts of histone-bound anthracycline. As Fig. 3 shows, Dauno-induced eviction of histones was augmented in the nuclei co-treated with Cis-Pt. Thus, the decreased nuclear presence of Dauno in the Cis-Pt co-treated samples (see Fig. 2b) is possibly due to the augmented histone release. Cis-Pt is known to cause distortions in the DNA structure which may be an important factor in its influence on Dauno-induced histone eviction: DNA containing a Cis-Pt 1, 2-intrastrand d(GpG) adduct is bent by 60° toward the major groove^37^. Eviction occurs also when live cells are treated with anthracyclines in line with references^9,12^ and it is also augmented by Cis-Pt co-treatment as shown in Fig. 3b. The Cis-Pt elicited increase in the eviction of H2B was detected already at low, while H3 eviction was augmented only at higher Dauno concentrations. Thus, interaction of the two drugs in terms of histone eviction appears to be synergistic in the whole concentration range of the anthracycline. In summary, Cis-Pt augments the binding of anthracyclines to the genomic DNA in spite of the overall decrease of cellular uptake in its presence (Figs. 2 and S3). In view of the increased histone eviction in the presence of Cis-Pt (Fig. 3) this may be a consequence of the appearance of DNA regions lacking topological constraints (restricting intercalation). Cis-Pt-augmented histone eviction was observed in HeLa cells at low as well as high Dauno concentration, and Cis-Pt in HeLa cells increased the DNA-binding of Dauno applied at low concentration (Fig. 2), formally ruling out the possibility that these factors might play a role in the antagonism found in HeLa cells at low Dauno concentration. On the other hand, the augmented formation of covalent Dauno-DNA adducts in the case of treatment with a combination of Dauno and Cis-Pt (Fig. S3) may contribute to the synergistic toxicity of the two drugs.

Regarding the effect of Dauno on Cis-Pt-DNA adduct levels, total platination was not affected (Fig. 5a), while the level of ICLs, assessed using a modified version of the alkaline renaturation assay (Fig. 4; see also Materials and Methods), was significantly decreased (Fig. 5b). The decreased ICL levels observed at low Dauno concentration were not further changed using higher concentrations of the anthracycline; thus, the antagonistic effect of Dauno on ICL formation appears to be similar in the entire Dauno concentration range tested. Therefore, if ICLs significantly contribute to Cis-Pt toxicity, Dauno co-treatment could mitigate these consequences in the low Dauno concentration range, while the synergistic other effects may overrule this at higher concentrations. The decrease of ICL formation is likely due to relaxation of negative supercoiling of the chromatin loops, in line with reference^20^ and with data on psoralen cross-linking^21^. Based on the above, the antagonism of low Dauno concentration with Cis-Pt in HeLa cells is suggestive of the importance of ICL formation in the cytotoxicity of Cis-Pt in certain experimental and treatment scenarios.

We have set up a model system comprising isolated nuclei treated with moderate and high salt concentration, to evict nucleosomes and elicit topological changes of the DNA in a controlled manner and in the absence of topological changes induced directly by intercalators. The LA-ICP-MS approach let us obtain absolute numbers for the total adduct levels and for ICLs and these are comparable to the numbers determined in other studies^17^. The magnitude and direction of changes in total platination levels (mainly intrastrand cross-links^38^) and ICLs were comparable to the changes observed when nuclei were treated with Cis-Pt and Dauno (Fig. 5). The Eviction of nucleosomes by salt and Dauno alike increases the target size for Cis-Pt binding. Furthermore, intrastrand crosslinks that involve bending of the DNA^22,23^ must be severely antagonized by any constrainment of its structure and augmented by the release of such constraints. On the other hand, the increase of free DNA regions upon nucleosome eviction is expected to favor formation of highly constrained, hyperplectonemic structures likely to occur in the case of long superhelical DNA molecules^39^. These antagonistic effects can explain the changes observed in total Cis-Pt-DNA adduct formation in our model system (Fig. 6). The increase of target size upon histone eviction may be dominant at moderate salt concentration, increasing adduct levels, while the hyperplectonemic constrainments in the naked DNA produced by high salt pre-treatment may prevent the bending of DNA required for intrastrand crosslinking^22,23^. On the other hand, ICL formation is strongly favored by negative Tw^20^. The superhelicity of the linker regions is generally assumed to be negative^40^. An ~30% of the overall linking number difference is estimated to partition as twist^41^. The nucleosomal structure entails slightly overwound DNA (Tw = 10.3 bp/turn compared to 10.5 bp/turn for B-DNA^42^. The negative twist in the linker regions together with the slightly positive twist inside the nucleosomes can account for the linker-preference of ICL formation^16^. At moderate salt treatment, upon eviction of H2A and H2B^12^, the enlarged nucleosome-free regions are expected to adopt a topology with higher degree of freedom to redistribute twist and writhe. As a consequence, Wr likely becomes more, and Tw less negative in the widening linker regions, in comparison with their state before eviction. Such changes would lead to a decrease in ICL numbers after treatment with 1.1 M salt. At high salt, upon complete eviction, the hyperplectoneme imposed topological constraints would antagonize proper apposition of the bases involved in ICL formation, further decreasing the chances of their generation. The data obtained in this model system are in line with the interpretation that the Dauno evoked decrease of ICL formation observed in isolated nuclei (Fig. 5) is, at least partly, due to Dauno induced eviction of the histone dimers (Fig. 3). To what extent Dauno-elicited Tw relaxation directly contributes to a decreased ICL formation could not be determined because nucleosome eviction necessarily accompanies intercalation. The scenario of complete eviction modeled by high salt concentration pre-treatment of isolated nuclei does not occur even at high Dauno concentrations. However, eviction may not be uniform along the genome, affecting certain chromatin regions more than others. Local stretches of naked DNA may be present when live cells are treated with Dauno. The formation of ICLs is expected to be reduced in these regions based on the data of Fig. 6. In summary, histone eviction by itself is expected to diminish ICL formation, while total adduct formation may not change due to the complexity of effects involving increased target size represented by a more relaxed naked DNA on the one hand and constraints emerging in the wake of DNA-DNA interactions upon hyperplectoneme formation on the other. These observations on the model system are in line with the effect of Dauno on total Cis-Pt adduct formation and on ICL formation (Fig. 5).

Since both drugs have multiple targets in the cell, antagonism as well as synergy could involve multitudes of factors. However, as both drugs are considered to primarily target the chromatin^15,35^, their interactions here may be important determinants of the pharmacological outcome of co-treatment. It remains to be determined if inhibition of Top II by Dauno, which was initially perceived as the primary cytotoxic effect of anthracyclines^43^, influences Cis-Pt toxicity. However, apoptosis induction by the DNA-Dauno adducts rather than Top II inhibition appears to be the crucial factor in the cytotoxicity of anthracyclines^44^.

In conclusion, as summarized in Supplementary Fig. S6, Dauno and Cis-Pt *antagonize* one another through a decrease of ICLs in the presence of Dauno (at its low and high cc. alike), and also via the diminished Dauno uptake in the presence of Cis-Pt. They could *synergize* with each other through enhanced histone eviction by Dauno in the presence of co-treatment with Cis-Pt, accompanied by an increment of DNA bound anthracycline. These drug interactions have not been described before to the best of our knowledge and may impact cytotoxicity reached by combination treament regimens *in vivo*.

## Materials and Methods

All reagents were purchased from Sigma-Aldrich (St. Louis, Missouri, USA), unless otherwise stated.

### Cells, treatments, cytotoxicity assay

HeLa cells expressing H2B-GFP or H3-GFP, control HeLa and Jurkat cells were cultured in DMEM or RPMI1640, respectively, supplemented with 10% FCS, 2 mM L-glutamine, 100 μg/ml streptomycin, 100 U/ml penicillin (both from Gibco, Grand Island, NY) in T-150 tissue culture flasks (Corning Glass Works, Corning, NY) in 5% CO_2_. Treatment with cytotoxic agents was performed in 24-well plates. Dauno (Pfizer, Budapest) was used in a concentration range of 0–15 μM. Cis-Pt (Accord-UK Ltd, United Kingdom, purchased as 1 mg/ml stock solution) was applied at 0–80 μM. For median effect analyses, Cis-Pt was added to the cells at 40 μM final concentration together with a concentration series of Dauno, for 16 hours. Cytotoxicity was measured based on mitochondrial function, assessed using the Resazurin based assay^45^. 100 μl aliquots of the drug treated cells were resuspended in colorless media and added to 96-well flat-bottom microplates to 20,000 cells per well. For each drug combination four parallel wells were prepared. 100 μl of freshly prepared Resazurin solution in colorless medium was added to each well to a final concentration of 18 µM. The plates were incubated at 37 °C for 24 h and fluorescence signals were measured at an excitation wavelength of 560 nm and an emission wavelength of 590 nm using Synergy H1 microplate reader (BioTek). Viability was expressed in the experimental groups in relation to untreated cells (control). The average concentrations of four wells were determined per dose. To determine synergism, additivity or antagonism, median effect analysis was performed using the method of Chou-Talalay^46^, using CompuSyn ver. 1.0 (ComboSyn Inc., Paramus, NJ, USA). The CI (combination index) reflects synergism, additivity or antagonism when CI < 1; CI = 1 or CI > 1, respectively. All indicated values are the averages of at least three independent experiments. The method involves recording of dose-response curves for the individually applied drugs, and measurement of the cytotoxic effect at a fixed dose of one drug, varying the concentration.of the other. Typical dose response curves are demonstrated in Fig. S1.

### Measurement of Dauno

#### Determination of total intracellular Dauno

In order to assess Dauno fluorescence in live cells and in isolated nuclei by flow-cytometry, HeLa cells were treated with 1.5 μM of Dauno alone or in combination with 40 μM of Cis-Pt for 16 hours. After trypsinization and resuspension of the cells, Dauno fluorescence intensity distributions were recorded in a flow-cytometer.

#### Determination of nuclear Dauno fluorescence

Aliquots of the drug treated cells above were suspended in 500 µl PBS (150 mM NaCl, 3.3 mM KCl, 8.6 mM Na_2_HPO_4_, and 1.69 mM KH_2_PO_4_, pH 7.4) and lysed in 500 μl ice-cold 0.2% (v/v) Triton X-100 dissolved in 1 × PBS/EDTA (5 mM EDTA in PBS), for 20 minutes. After lysis, nuclei were washed twice with 4 ml ice cold PBS/EDTA then resuspended in 1 ml PBS/EDTA and Dauno fluorescence intensity distributions were recorded by flow-cytometry.

#### Determination of DNA-bound Dauno via Hoechst-quenching

HeLa cells were treated with 1.5 μM of Dauno alone or in combination with 40 μM of Cis-Pt for 16 hours, then, without washing the samples, stained with 35 μM Hoechst 33342 for 30 minutes. After trypsinization, the cells were analysed by flow-cytometry. For flow-cytometry set up, refer to supplementary methods.

### Measurement of histone eviction

Histone eviction was measured by the QINESIn assay developed in our lab^12^. Briefly, the agarose-embedded GFP-histone expressor cells at the bottom of ibidi wells were permeabilized with 500 μl ice cold 1% (v/v) Triton X-100 dissolved in 1 × PBS/EDTA, washed with 500 μl ice cold 1 × PBS/EDTA and these samples of nuclei were treated with different concentrations of Dauno. The histones remaining chromatin-bound were determined cell-by-cell using LSC scans (see below) of the samples.

### Measurement of ICLs and total Cis-Pt-DNA adduct levels by LSC

#### Preparation of comets and halos

Following exposure to drugs, the cells were centrifuged at 175 g for 5 min at 4 °C, then resuspended to a final concentration of 1 × 10^6^ cells/ml in PBS, kept at 37 °C and mixed at a v/v ratio of 1: 3 with 1% Low melting point agarose dissolved in PBS. 100 μl of the cell-agarose suspension was pipetted onto Superfrost microscope slides (Thermo Fisher Scientific, Waltham, Massachusetts, USA) and were covered with coverslips (VWR, Hungary, 60 mm × 24 mm). The slides were pre-coated by submerging them in molten 1% agarose (SeaKem^®^ LE Agarose, Lonza) dissolved in water, and then allowed to dry overnight at room temperature. The cells were left to sediment on the surface of the slides for 4 minutes at 37 °C, then kept on ice for 5 min. After polymerisation of the agarose, the coverslips were gently removed, then the slides were submerged in glass staining tanks containing ice-cold PBS/EDTA for 5 minutes, then submerged in ice-cold TRIS/EDTA (Tris-HCl 20 mM pH = 7.5, 5Mm EDTA), 150 mM NaCl and 0.1% v/v Triton X-100 for 10 minutes, next in PBS/EDTA containing 1% Triton X-100 for another 10 minutes. After washing in PBS/EDTA for 10 minutes, the samples were equilibrated with nickase buffer (10 mM Tris-HCl pH 8.0, 50 mM NaCl, 10 mM MgCl_2_, 1 mg/ml BSA), then nickase treatment was performed covering the slides with 500 μl of nickase buffer containing the frequent cutter nicking endonuclease Nt.CviPII (New England Biolabs Inc., Ipswich, MA, USA) at a final concentration of 0.001 U/ml and incubated in a wet chamber at 37 °C, for 20 min. After washing with ice-cold PBS/EDTA three times for 10 minutes, the slides were submerged in freshly made alkaline lysis buffer (2.5 mM NaCl, 1% N-Laurylsarcosine sodium salt, 10 mM Tris, 150 mM NaOH, 100 mM EDTA, 10% DMSO, and 1% Triton X-100; pH = 10) at room temperature, for 1 h. After alkaline denaturation, the slides were placed in an electrophoresis tank (Cleaver Scientific Ltd, United Kingdom) filled with alkaline running buffer (300 mM NaOH, 1 mM EDTA, pH = 13). After electrophoresis (for 20 min at 200 mA), the samples were neutralized with 0.4 M Tris (pH = 7.5) for 10 min before staining with SYBR Green I (Thermo Fisher Scientific, Waltham, Massachusetts, USA), 1: 5,000 diluted in PBS/EDTA. Since no background fluorescence was detected following S1 digestion (see Fig. 4), simple intensity measurements instead of calculating tail-moments sufficed, so the steps related to comet formation (nickase digestion and electrophoresis) were omitted and the experiments were performed in ibidi 8-wells microslide. These halo preparations allowed comparison of samples in ibidi slides that proved to be more convenient and reliable, improving also throughput.

#### Detection of ICLs

Non-renatured DNA of the comets and halos prepared as above were removed by S1 nuclease digestion. The samples were equilibrated with S1 buffer (280 mM NaCl, 50 mM CH_3_COONa and 4.5 mM ZnSO_4_, pH = 4.4) for 30 min, then the samples were covered with 500 µl of S1 enzyme solution. The enzyme (Thermo Fisher Scientific, Waltham, MA, USA) was diluted to 1000 U/ml concentration in S1 nuclease buffer in the working solution. The samples were incubated on ice for 5 min with the enzyme for equilibration, then digestion was performed at 37 °C, for 20 min. After washing in PBS/EDTA, the samples were stained with SYBR Green I as described above, for 1 h.

#### Determination of total amount of Cis-Pt-DNA adducts

Nuclear halos were labeled by indirect immunofluorescence using anti-Cisplatin primary antibody as described below. Agarose-embedded nuclei on ibidi slides were exposed to the combinations of Cis-Pt and Dauno at 4 °C for 16 hours, washed with ice cold PBS/EDTA three times for 5 minutes each, treated with Proteinase K (Thermo Fisher Scientific, Waltham, Massachusetts, USA) dissolved in 0.4 M EDTA, 1% Sodium lauroyl sarcosinate, 10 mM Tris, pH = 8 at 0.5 mg/ml final concentration, at 42 °C, for 24 h. The enzymatic reaction was stopped by incubating the samples with 1:10 dilution of 10 mM PMSF) at room temperature, for 10 minutes. Then the samples were denatured and renatured as described above; electrophoresis was skipped. Immunofluorescence labeling was performed at 4 °C, overnight, using rat monoclonal anti-Cisplatin modified DNA antibody (Abcam, Cambridge, UK; 1 mg/ml) diluted at a titer of 1:800 in 150 µl of PBS/EDTA/1% BSA. Samples were washed 6 times for 10 min each, followed by incubating the samples with 500 μl 5% (m/v) Blotto Non-Fat Dry Milk (Santa Cruz Biotechnology Inc., Santa Cruz, California, USA) in PBS/EDTA on ice for 30 min. Labeling with the secondary antibody was performed using Alexa Fluor 488 (A488) conjugated Donkey anti-rat IgG (Thermo Fisher Scientific, Waltham, Massachusetts, USA), at a titer of 1:800, diluted in PBS/EDTA/3% BSA, from 2 mg/ml stock solutions. After labeling with the secondary antibody, nuclei were washed with 500 μl ice-cold PBS/EDTA 3 times for 10 min each. Then the samples were fixed using 1% formaldehyde at 4 °C, overnight. After fixation, the nuclei were washed with 500 µl ice-cold PBS/EDTA three times for 5 min and fluorescence intensity distributions were recorded by LSC. For studying the effect of salt induced histone eviction on total Cis-Pt-DNA adduct formation (Fig. 6a), the agarose embedded nuclei of HeLa cells were prepared on the slides as described above and were treated with 1.1 M or 1.6 M of NaCl in PBS/EDTA 6 times for 10 min each and subsequently washed with PBS/EDTA. These samples were exposed to 40 µM of Cis-Pt, for 16 hr, then treated with proteinase K followed by PMSF and washing steps, then labelled by immunofluorescence for the LSC measurements, as described above. Then the same samples were dehydrated with an ascending series (20–95%) of ethanol for subsequent LA-ICP-MS analysis.

### Laser scanning cytometry (LSC)

Automated microscopic imaging was performed using an iCys instrument (iCys® Research Imaging Cytometer; CompuCyte, Westwood, MA, USA). Green fluorescent protein (GFP), SYBR Green, A488, and propidium iodie (PI) were excited using a 488 nm Argon ion laser. A647 was excited with a 633 nm HeNe laser. The fluorescence signals were collected via an UPlan FI 20 × (NA 0.5) objective. GFP and A488 and SYBR Green fluorescence were detected through 510/21 nm, 530/30 nm and 550/30 nm filters respectively, while A647 and PI were detected through a 650 nm/LP filter. Each field (comprising 1000 × 768 pixels) was scanned with a step size of 1.5 μm. Data evaluation was performed using the iCys 7.0 software for Windows XP. Gating of G1 phase cells was based on the fluorescence intensity distribution curves of the DNA stained with PI.

### Determination of Pt in the nuclei of agarose-embedded cells using quantitative laser ablation-inductively coupled plasma-mass spectrometry spot analysis

#### LA-ICP-MS instrumentation

An Analyte G2 193 nm ArF* excimer-based LA-unit (Teledyne Photon Machines Inc., Bozeman, MT, USA), equipped with a low-dispersion Cobalt ablation cell, was coupled to a quadrupole-based Agilent 7900 ICP-mass spectrometer (Agilent Technologies Inc., Tokyo, Japan) *via* the ARIS (Aerosol Rapid Introduction System, Teledyne Photon Machines Inc., Bozeman, MT, USA), providing fast aerosol transport^47–49^. For preparation of external calibration standards, instrument settings and data acquisition conditions see supplementary methods and supplementary Table 1.

#### Spot analysis

The determination of Pt in the nuclei of agarose-embedded cells was performed using a quantitative LA-ICP-MS spot analysis approach, derived from the single-cell LA-ICP-MS analysis method described in a previous work by Van Acker *et al*.^50^. See supplementary methods for details.

## Supplementary information

Supplementary Material.
