# Author Correction: Interactions of Cisplatin and Daunorubicin at the Chromatin Level

**DOI:** 10.1038/s41598-020-60515-3

**Published:** 2020-02-26

**Authors:** Erfaneh Firouzi Niaki, Thibaut Van Acker, László Imre, Péter Nánási, Szabolcs Tarapcsák, Zsolt Bacsó, Frank Vanhaecke, Gábor Szabó

**Affiliations:** 1 Department of Biophysics and Cell Biology, University of Debrecen, Faculty of Medicine, Debrecen, H-4032 Hungary; 2 Department of Chemistry, Atomic & Mass Spectrometry – A&MS Research Unit, Ghent University, Campus Sterre, Krijgslaan 281-S12, 9000 Ghent, Belgium

Correction to: *Scientific Reports* 10.1038/s41598-020-57702-7, published online 24 January 2020

This Article contains errors in Reference 41 which was incorrectly given as:

*in eLS*.

The correct reference is listed below as ref. 1:
