# Supplementary material for: Interactions of Cisplatin and Daunorubicin at the Chromatin Level
Source: Sci Rep. 2020 Jan 24;10:1107. doi: 10.1038/s41598-020-57702-7 (PMC6981277; doi:10.1038/s41598-020-57702-7)
Supplement: Supplementary file 1 — Supplementary Material. [file 41598_2020_57702_MOESM1_ESM.docx]

**Supplemantary information**

**Interactions of Cisplatin and Daunorubicin at the chromatin level**

Erfaneh Firouzi Niaki^1^, Thibaut Van Acker^2^, László Imre^1^, Péter Nánási Jr^1^,

Szabolcs Tarapcsak^1^, Zsolt Bacso^1^, Frank Vanhaecke^2^ and Gábor Szabó^1*^

^1^ *Department of Biophysics and Cell Biology, University of Debrecen, Faculty of Medicine Debrecen, H-4032, Hungary*

^2^ *Department of Chemistry, Atomic & Mass Spectrometry – A&MS Research Unit, Ghent University, Campus Sterre, Krijgslaan 281-S12, 9000, Ghent, Belgium*

*Corresponding author: szabog@med.unideb.hu, 0036304463955

**Supplementary methods:**

***Preparation of external calibration standards:*** Homogeneously dried gelatin droplets (0, 10, 50 and 200 µg g^-1^ Pt) were prepared as external calibration standards, following the protocol described by Sala *et al* [[1](#_ENREF_1)]*.* Gelatin standard solutions (10% w/v) were prepared by mixing gelatin powder (VWR International, Leuven, Belgium) with ultra-pure Milli-Q water (resistivity ≥ 18.2 MΩ cm), obtained from a Direct-Q3 water purification system (Millipore, Molsheim, France) and spiked with varying concentrations of Pt (from 1 g L^-1^ Pt stock solution in 0.7 M HCl, Inorganic Ventures, Christiansburg, VA, USA). Subsequently, melting of the gelatin powder was achieved by placing the gelatin standard solutions in a laboratory water bath at 60 °C. After proper mixing of the gelatin solutions using a vortex system (VWR International, Leuven, Belgium), gelatin droplets of 20 mg were deposited onto a Superfrost^™^ glass microscope slide (Thermo Fisher Scientific, Waltham, MA, USA) using a micropipette and dried under controlled conditions (closed Petri dish, 1 h at 95 °C) in a mechanical convection oven to achieve highly homogeneous gelatin droplets.

***Spot analysis*:** A circular laser spot size of 10 µm diameter was selected to quantitatively ablate 100 individual cell nuclei per sample by firing 4 consecutive laser pulses at 250 Hz and the resulting ^195^Pt signal peaks were individually integrated after background signal subtraction. The dried gelatin droplet standards were spot ablated prior to analysis of the samples using the same instrument settings and data acquisition conditions.

External calibration curves were constructed based on the Pt concentrations and integrated ^195^Pt signal intensities. The slope of the calibration curve corresponds to the sensitivity $A$ (counts/µg g^-1^) and the following equation was used to calculate the average number of Pt atoms per 100 kbp of DNA $\overline{N}_{Pt}$:

$$\overline{N}_{Pt}=\left( \frac{\overline{x}\cdot\rho\cdot V\cdot N_{A}}{A\cdot M\cdot N_{bp}} \right)\cdot{10}^{-13}$$

With the volumetric mass density $\rho$ of the nuclei embedded in agarose (g cm^-3^), the ablated volume $V$ (µm^3^), Avogadro’s constant $N_{A}$ (mol^-1^) and the total number of base pairs of DNA per cell nucleus $N_{bp}$ (-).

**Tuning of the LA-ICP-MS instrumentation:** Daily tuning of the instrument settings for high sensitivity across the elemental mass range (^7^Li^+^, ^115^In^+^ and ^238^U^+^), low laser-induced elemental fractionation (^238^U^+^/^232^Th^+^ ≈ 1), low oxide formation (^238^U^16^O^+^/^238^U^+^ < 1%) and low gas background (^15^N^16^O^+^) was performed while ablating NIST SRM 612 glass certified reference material (National Institute for Standards and Technology, Gaithersburg, MD, USA).

**Flow-cytometry:** A Becton Dickinson FACSAria III Cell Sorter (Becton Dickinson, Mountain View, CA, USA) was used. Dauno was excited using the 488 nm line of a solid state laser and the emitted light was detected using a 695/40 nm band-pass filter. Hoechst 33342 fluorescence was measured by excitation at 375 nm and emission detection using a 450/50 nm band-pass filter. Fluorescence signals were collected in logarithmic mode and the flow-cytometric data were analyzed by the ReFlex software [[2](#_ENREF_2)].

**Measurement of histone eviction:** Histone eviction was measured as described in a previous work from Imre *et al* [[3](#_ENREF_3)]. Briefly, H2B-GFP and H3-GFP expressor cells were embedded in agarose layers in wells of 8-well chambers (Ibidi, Martinsried, Germany) so that each well contained 10 - 30,000 cells per well, in PBS. In the case of H2B-GFP HeLa, the cells were lysed by adding 450 μl ice cold 1% (v/v) Triton X-100 dissolved in PBS/EDTA to the wells. This step was repeated once more then the embedded nuclei were washed twice before being exposed to a concentration series of Dauno +/- Cis-Pt dissolved in PBS, at 4 ^o^C, for 16 h. In the case of H3-GFP HeLa, the cells were treated with Dauno +/- Cis-Pt in complete medium at 37 ^o^C for 16 h, before embedding. The samples were fixed in 1% formaldehyde (dissolved in 1 × PBS/EDTA) containing 12.5 μg/ml propidium iodide (PI), at 4 °C, overnight. The stained nuclei were washed and the fluorescence intensity distributions were recorded using an iCys laser scanning cytometer (LSC). Analysis of the elution curves was performed using SigmaPlot 11.0 software, using the Four Parameter Logistic curve-fitting subroutine.

**Urea-agarose gel electrophoresis:** The method of Materna et al., further developed by us [[4](#_ENREF_4),[5](#_ENREF_5)], was used to compare the genomic DNA of Jurkat cells treated with Dauno alone, with a combination of Dauno and Cis-Pt and the untreated control cells. Further details are described Fig. S3. The „dimming” of the EBr fluorescence in the Dauno-treated samples was indicative of covalent Dauno-DNA adducts

**Supplementary Tables and Figures**

**Supplementary Table. 1**

Instrument settings and data acquisition conditions for spot analysis *via* LA-ICP-MS.

| **Teledyne Photon Machines Analyte G2 193 nm ArF* excimer LA system + Cobalt ablation cell + Aerosol Rapid Introduction System (ARIS)** | |
| --- | --- |
| Energy density ($J cm^{-2}$) | 0.44 |
| Repetition rate ($Hz$) | 250 |
| Beam waist diameter ($\mu m$) | 10 (circle) |
| Dosage ($shots {position}^{-1}$) | 4 |
| He carrier gas flow rate ($L {min}^{-1}$) | 0.3 |
| **Agilent 7900 ICP-MS unit** | |
| RF power ($W$) | 1500 |
| Sampling depth ($mm$) | 5.5 |
| Ar plasma gas flow rate ($L {min}^{-1}$) | 15 |
| Ar auxiliary gas flow rate ($L {min}^{-1}$) | 0.9 |
| Ar make-up gas flow rate ($L {min}^{-1}$) | 1.01 |
| Acquired m/z ratio (*amu*) | 195 (^195^Pt) |
| Respective dwell time ($ms$) | 1 |
| Total scan cycle time ($ms$) | 1 |

**Supplementary Figure 1.**


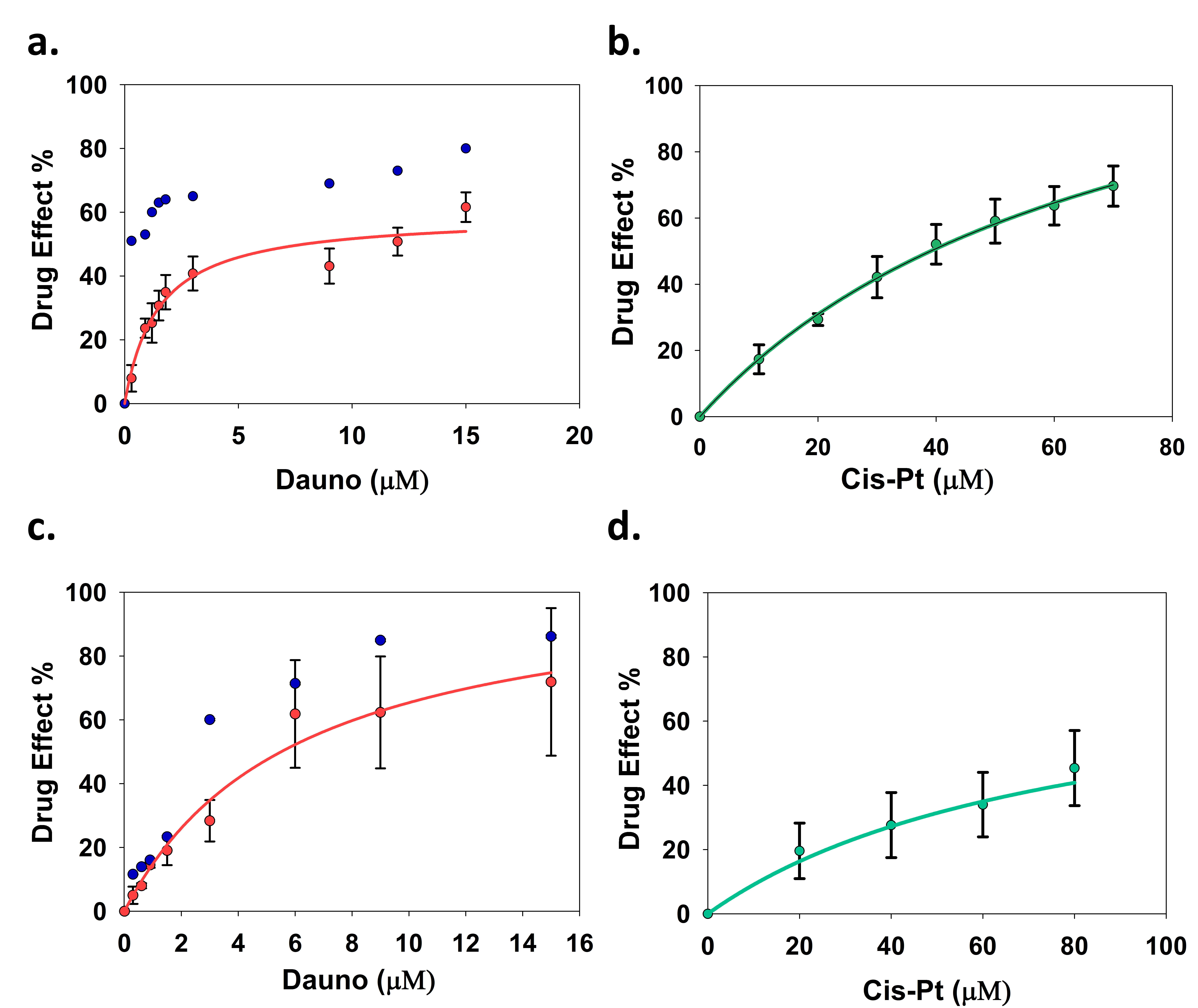
**Fig. S1**

**Dose response curves from average data of several experiments**

Jurkat cells were treated with Dauno (red line; panel **a**) or Cis-Pt (green line; **b**) in a concentration range of 0.3-15 μM and 10-70 µM, respectively. Averaged dose-response curves of 4 independent samples (a triplicate and an independent biological experiment). Panels **c** and **d**: HeLa cells treated with Dauno and Cis-Pt at a concentration range of 0.3-15 μM and 20-80 µM, respectively. Blue circles: dose-effect values of treatment with Dauno in combination with 40 µM Cis-Pt. Averaged dose-response curves of 4 independent experiments. Error bars represents SDs.

**Supplementary Table. 2**

**
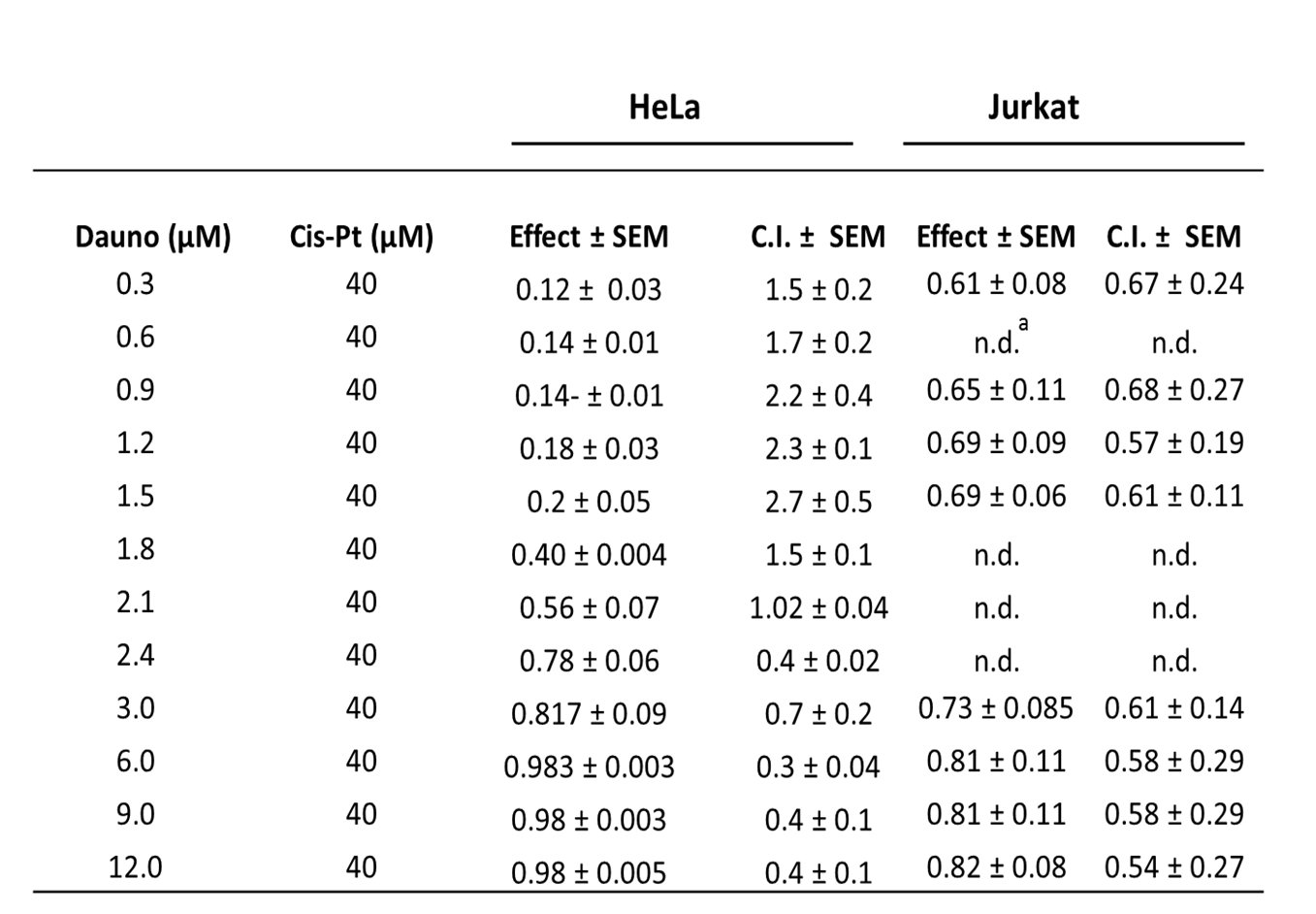
**

^a^ n.d. indicates no data

**Drug effect and corresponding C.I. values of Fig. 1**

**Supplementary Figure 2.**

**
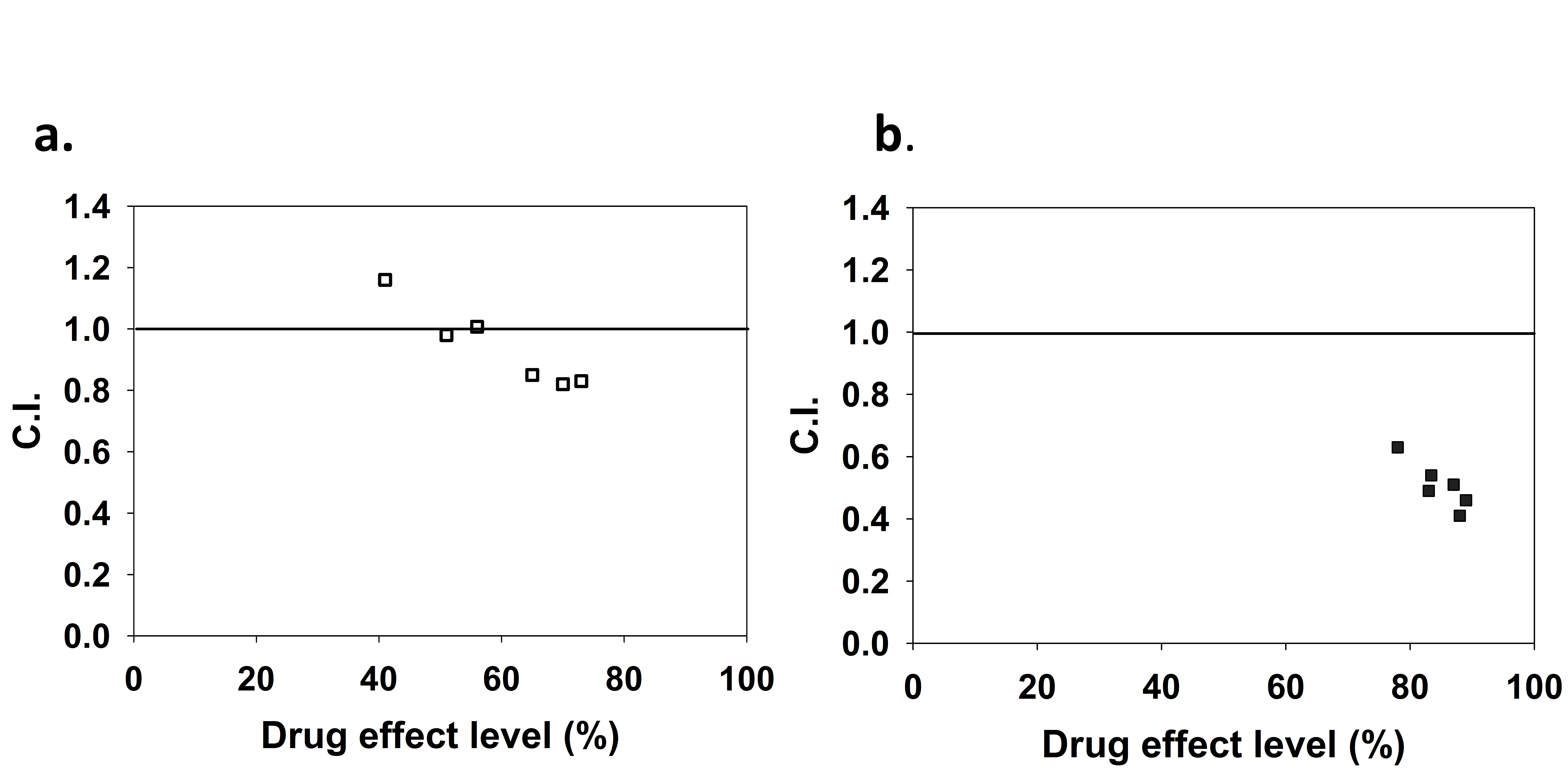
**

| Dauno (μM) | CisPt ( μM) | Drug effect level ± SD | C.I. |
| --- | --- | --- | --- |
| 18 | 10 | 0.78 ± 0.04 | 0.63 |
| 18 | 20 | 0.83 ± 0.05 | 0.49 |
| 18 | 30 | 0.834 ± 0.05 | 0.54 |
| 18 | 40 | 0.87 ± 0.05 | 0.41 |
| 18 | 50 | 0.89 ± 0.05 | 0.51 |
| 18 | 60 | 0.92 ± 0.06 | 0.46 |

| Dauno (μM) | CisPt ( μM) | Drug effect level ± SD | C.I. |
| --- | --- | --- | --- |
| 3 | 10 | 0.415 ± 0.03 | 1.16 |
| 3 | 20 | 0.519 ± 0.09 | 0.98 |
| 3 | 30 | 0.567 ± 0.06 | 1.00 |
| 3 | 40 | 0.657 ± 0.06 | 0.85 |
| 3 | 50 | 0.708 ± 0.06 | 0.82 |
| 3 | 60 | 0.735 ± 0.06 | 0.83 |

**Fig. S2**

**Dependence of C.I. values on Cis-Pt concentration**

C.I. values determined for Jurkat cells treated with 3 μM Dauno (**a**) and 18 μM Dauno (**b**) in combination with Cis-Pt varied between 10 and 60 μM. The tables below the graphs show the specific C.I. values plotted.

**Supplementary Figure 3.**

**
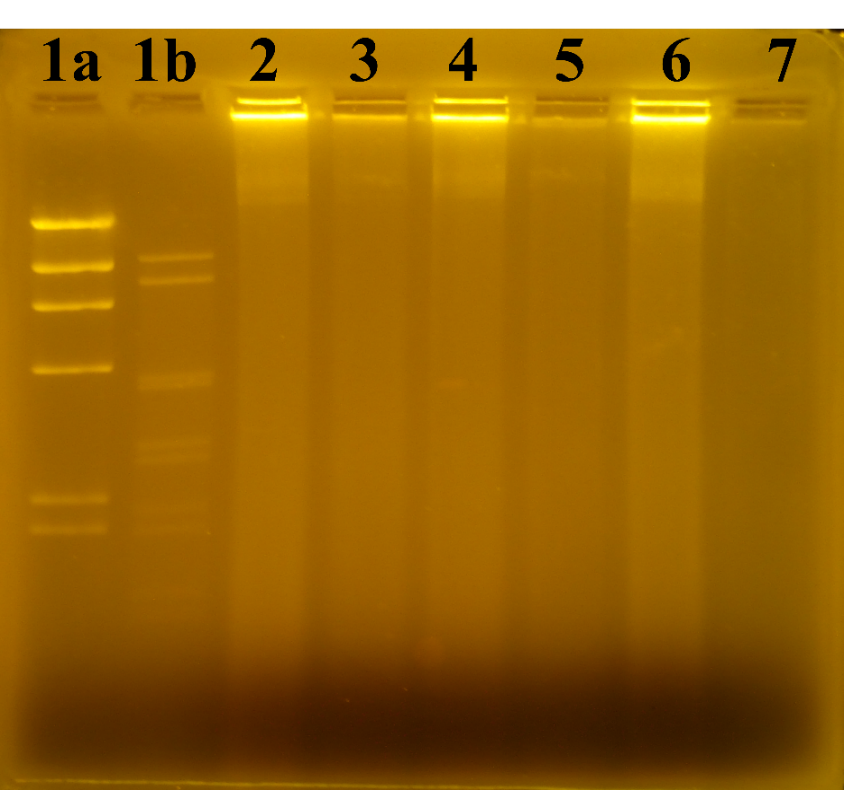
**

|  | **2** | **3** | **4** | **5** | **6** | **7** |
| --- | --- | --- | --- | --- | --- | --- |
| **Dox** | ***-*** | ***+*** | ***-*** | ***+*** | ***-*** | ***+*** |
| **Pt** | ***-*** | ***-*** | ***+*** | ***+*** | ***++*** | ***++*** |

**Fig. S3**

**DNA binding of Dox is augmented when co-administered with Cis-Pt**

Jurkat cells were treated with 9 μM Dox, known to bind to the DNA covalently [[6](#_ENREF_6)], and 10 (+) or 25 μM (++) Cis-Pt for 16 hrs. The DNA of the samples was analyzed by urea-agarose gel electrophoresis, as described in [[4](#_ENREF_4)] . Briefly, the cells were embedded into LMP-agarose plugs, deproteinized and treated with the Nt.CviPII nickase enzyme. This was necessary to allow the entry of part of the DNA, after heat-denaturation, into the gel. The samples of agarose plugs were equilibrated with 9 M urea-containing loading buffer, denaturated at 90 ^o^C for 5 mins, allowed to cool to RT then loaded onto an agarose gel also containing urea. The denatured fragments stay single-stranded in these conditions. After conventional electrophoresis, the gel was stained with 5 μg/ml EBr. The “dimming” of the lanes in the Dox-treated samples due to the presence of covalently bound anthracyclin is increasing by increasing Cis-Pt concentration. Lanes 1a,b: Lambda Hind III fragments run without (a) and with (b) heat-denaturation.

The uncropped, recolored to grayscale picture of the gel is depicted.

**Supplementary Figure 4.**

**
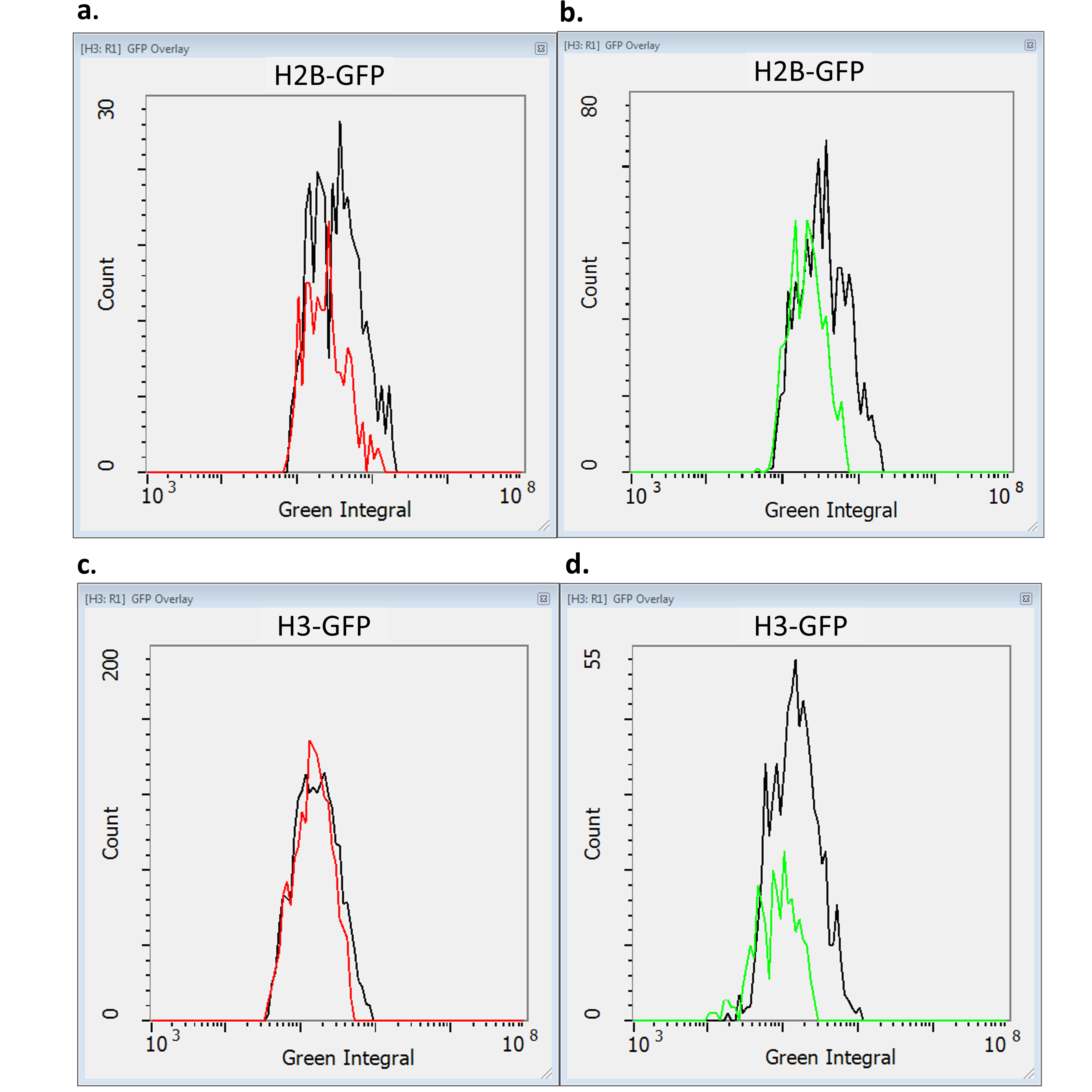
**

**Fig. S4**

**Fluorescence intensity distribution histograms of H2B-GFP and H3-GFP**

Fluorescence intensity distribution histograms of the LSC data shown in Fig. 3. GFP-H2B (a, b) and GFP-H3 (c, d) distributions obtained at 0 uM (black lines) and at the highest concentration of Dauno in the absence (red) and presence of Cis-Pt (green). Fluorescence is shown in log scale.

**Supplementary Figure 5.**


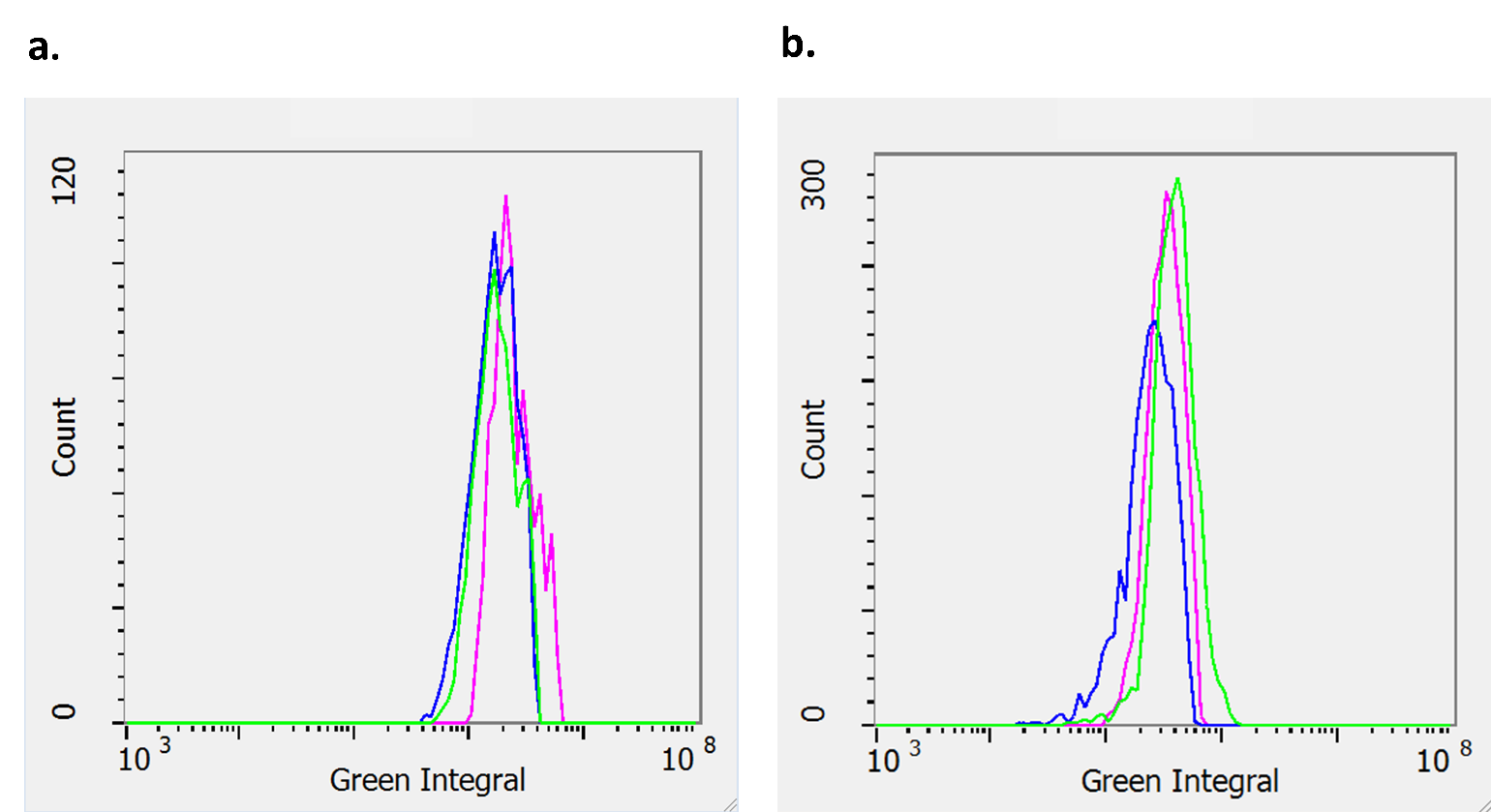


**Fig. S5**

**Fluorescence intensity distribution curves of Total Cis-Pt adduct and ICL**

Fluorescence intensity distribution histograms of the LSC data shown in Fig. 6. Panel **a** shows the total Cis-Pt adduct levels and corresponds to Fig. 6b, panel **b** represents ICLs and corresponds Fig. 6c. Green lines: 0 salt; pink lines: 1.1 M salt; blue lines: 1.6 M salt. Fluorescence is shown in log scale.

**Supplementary Figure 6.**


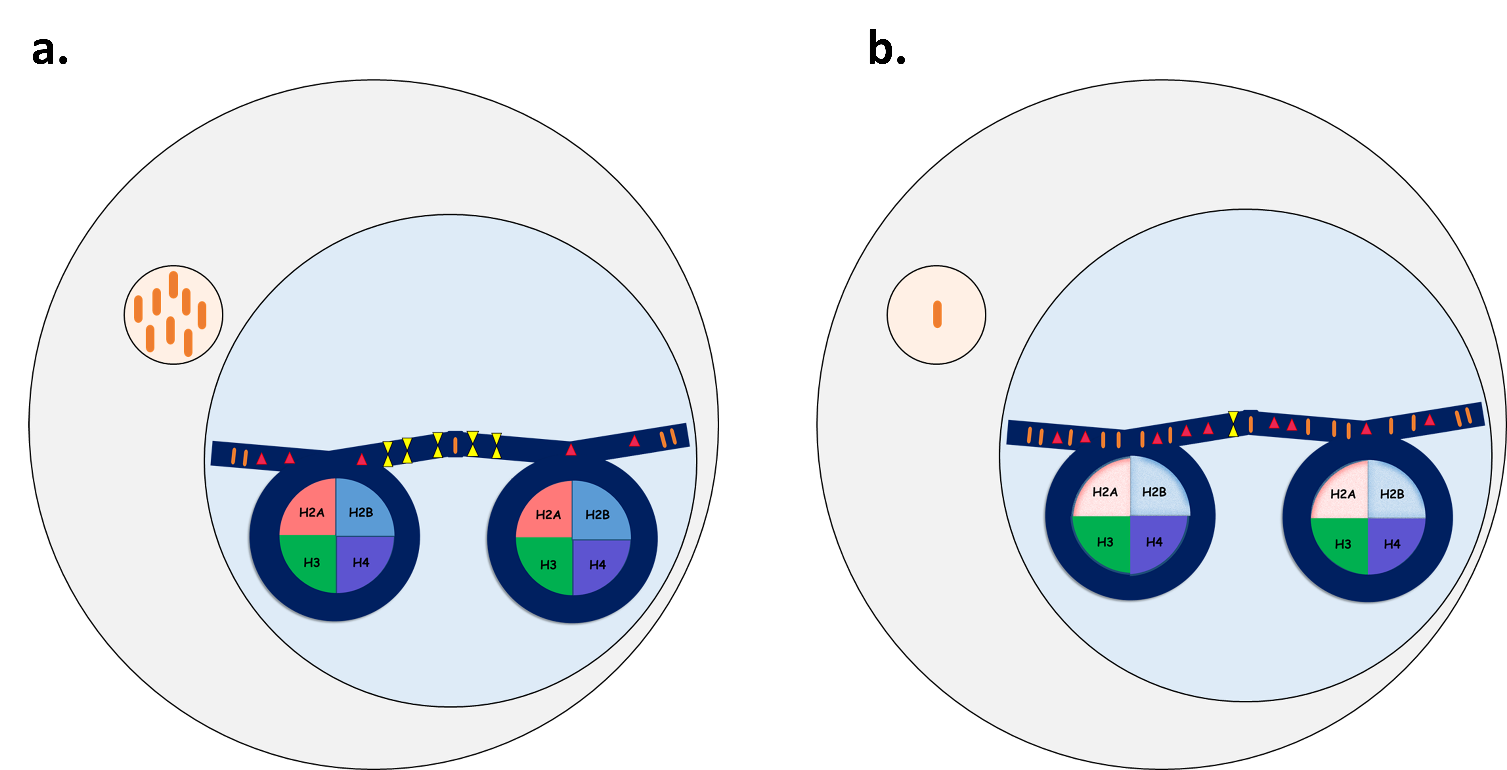


**Fig. S6**

**Schematic interpretation of the interactions observed.**

**a** **No influence on each other’s binding**

Dauno (orange rectangle) is present in cytoplasm mainly in the endosomal compartment. In the nucleus it is bound both to nucleosomes and intercalates into the DNA (blue). In DNA, Cis-Pt forms mainly intrastrand (red triangle) crosslinks and, less frequently, ICLs (yellow double triangles).

**b** **Mutual influence**

In the presence of Cis-Pt, Dauno concentration decreases in the cytoplasm (due to decreased uptake into the cell and/or the endosomal compartment). In the nucleus, its total amount is not affected by Dauno co-treatement, but the number of ICLs is decreased. The number of DNA bound Dauno is increased when Cis-Pt is co-administered. Histone eviction is represented by the lighter colors of the core histones that are readily released. Dauno molecules bound to the histones are not represented in the figure, they are assumed to be released from chromatin together with the histones they bind to.

1. Sala M, Selih VS, van Elteren JT (2017) Gelatin gels as multi-element calibration standards in LA-ICP-MS bioimaging: fabrication of homogeneous standards and microhomogeneity testing. The Analyst 142 (18):3356-3359. doi:10.1039/c7an01361b

2. Szentesi G, Horvath G, Bori I, Vamosi G, Szollosi J, Gaspar R, Damjanovich S, Jenei A, Matyus L (2004) Computer program for determining fluorescence resonance energy transfer efficiency from flow cytometric data on a cell-by-cell basis. Computer methods and programs in biomedicine 75 (3):201-211. doi:10.1016/j.cmpb.2004.02.004

3. Imre L, Simandi Z, Horvath A, Fenyofalvi G, Nanasi P, Niaki EF, Hegedus E, Bacso Z, Weyemi U, Mauser R, Ausio J, Jeltsch A (2017) Nucleosome stability measured in situ by automated quantitative imaging. 7 (1):12734. doi:10.1038/s41598-017-12608-9

4. Hegedus E, Kokai E, Kotlyar A, Dombradi V, Szabo G (2009) Separation of 1-23-kb complementary DNA strands by urea-agarose gel electrophoresis. Nucleic acids research 37 (17):e112. doi:10.1093/nar/gkp539

5. Materna T, Weber S, Kofler-Mongold V, Phares W (1998) Electrophoretic separation of both single- and double-stranded nucleic acids in the same urea-containing agarose gel. Analytical biochemistry 255 (1):161-163. doi:10.1006/abio.1997.2464

6. Yang F, Teves SS, Kemp CJ, Henikoff S (2014) Doxorubicin, DNA torsion, and chromatin dynamics. Biochimica et biophysica acta 1845 (1):84-89. doi:10.1016/j.bbcan.2013.12.002
